# Group membership, not diet, structures the composition and functional potential of the gut microbiome in a wild primate

**DOI:** 10.1128/msphere.00233-24

**Published:** 2024-06-28

**Authors:** Peter M. Finnegan, Paul A. Garber, Anna C. McKenney, Júlio César Bicca-Marques, María Fernanda De la Fuente, Filipa Abreu, Antonio Souto, Nicola Schiel, Katherine R. Amato, Elizabeth K. Mallott

**Affiliations:** 1 Department of Anthropology, Northwestern University, Evanston, Illinois, USA; 2 Department of Anthropology, University of Illinois Urbana-Champaign, Urbana, Illinois, USA; 3 Program in Ecology, Evolution, and Conservation Biology, University of Illinois Urbana-Champaign, Urbana, Illinois, USA; 4 International Centre of Biodiversity and Primate Conservation, Dali University, Dali, Yunnan, China; 5 Department of Natural Sciences, Parkland College, Champaign, Illinois, USA; 6 Escola de Ciências da Saúde e da Vida, Pontifícia Universidade Católicado Rio Grande do Sul, Porto Alegre, Rio Grande do Sul, Brazil; 7 Program in Ethnobiology and Nature Conservation, Paraíba State University, Campina Grande, Paraíba, Brazil; 8 Comparative BioCognition, Institute of Cognitive Science, University of Osnabrück, Osnabrück, Germany; 9 Department of Zoology, Federal University of Pernambuco, Recife, Pernambuco, Brazil; 10 Laboratório de Etologia Teórica e Aplicada, Department of Biology, Federal Rural University of Pernambuco, Recife, Brazil; 11 Department of Biology, Washington University in St. Louis, St. Louis, Missouri, USA; University of Michigan-Ann Arbor, Ann Arbor, Michigan, USA

**Keywords:** gut microbiota, *Callithrix jacchus*, common marmosets, sociality

## Abstract

The gut microbiome has the potential to buffer temporal variations in resource availability and consumption, which may play a key role in the ability of animals to adapt to a broad range of habitats. We investigated the temporal composition and function of the gut microbiomes of wild common marmosets (*Callithrix jacchus*) exploiting a hot, dry environment—Caatinga—in northeastern Brazil. We collected fecal samples during two time periods (July–August and February–March) for 2 years from marmosets belonging to eight social groups. We used 16S rRNA gene amplicon sequencing, metagenomic sequencing, and butyrate RT-qPCR to assess changes in the composition and potential function of their gut microbiomes. Additionally, we identified the plant, invertebrate, and vertebrate components of the marmosets’ diet via DNA metabarcoding. Invertebrate, but not plant or vertebrate, consumption varied across the year. However, gut microbiome composition and potential function did not markedly vary across study periods or as a function of diet composition. Instead, the gut microbiome differed markedly in both composition and potential function across marmosets residing in different social groups. We highlight the likely role of factors, such as behavior, residence, and environmental heterogeneity, in modulating the structure of the gut microbiome.

**IMPORTANCE:**

In a highly socially cohesive and cooperative primate, group membership more strongly predicts gut microbiome composition and function than diet.

## INTRODUCTION

A major ecological and evolutionary challenge for wild animals is adjusting to environmental changes in ways that ensure sufficient nutritional resources for survival and reproduction. While there is a rich body of literature exploring behavioral and physiological responses to environmental changes, such as variation in food availability, temperature, or rainfall, studies of the gut microbiota over the past two decades indicate that short-term changes in the composition of the gut microbiome allow animals to quickly adjust to seasonal differences in the nutritional content of their diet (1, 2). Therefore, variation in the diversity and composition of host-associated microbiomes is influenced by changes in the host’s environment (3–7) and can have a significant impact on host nutrition and health (8–12).

Wild animals inhabit ecosystems characterized by temporal variation in climate (temperature and rainfall) and food availability. For example, in some tropical forests, rainfall may be strictly seasonal and characterized by several months with almost no precipitation (dry season), followed by a period of several months of heavy precipitation (wet season) (13). Animals in these habitats are likely to experience environment-microbiome-health interactions in a cyclical, somewhat predictable way. Conversely, at other sites, rainfall may be fairly continuous throughout the year [i.e., Atlantic Forest (13)] or absent during most months of the year [i.e., Caatinga (13, 14)]. Depending on how these patterns affect the phenology of local plant communities, foragers are likely to experience extreme to minor seasonal variations in the types and amounts of food available to them (15, 16).

Temporal or seasonal changes in gut microbiome composition have been documented across animal species (17–32). These changes in gut microbiome composition may result in changes in function that compensate for nutrient deficiencies in the diet (1, 18, 29) and/or increase the ability of the host to extract energy from otherwise undigestible dietary items (21), particularly for animal populations occupying highly seasonal environments. However, while temporal shifts in the gut microbiome may buffer short-term environmental changes, social interactions between group members may be more important for maintaining the presence of host-associated microbes that are beneficial within a specific habitat or during a particular time of year (33, 34). In this regard, group membership has been shown to more strongly predict gut microbiome composition than temporal differences in diet in certain host species (17, 35–37). For example, group membership explained 18.6% of the variation in gut microbiome composition and 10.8% of the variation in gut microbiome function in wild baboons (*Papio cynocephalus*) (38). Similarly, gut microbiome composition in Verreaux’s sifakas (*Propithecus verreauxi*) was found to vary markedly across groups and was unrelated to temporal shifts in diet or group differences in diet (39). Intra- and intergroup similarity in microbiome composition may reflect the degree of horizontal transmission of microbes during social interactions and physical contact both within and between groups (33, 34, 38, 40, 41). For instance, hyenas (*Crocuta crocuta*) have group-specific microbiomes in their scent glands that produce odors important in chemical communication and social cohesion (42). Scent marking behavior in hyenas, where group members sequentially mark the same location, may contribute to these patterns of within-group similarity and between-group differences in microbiome composition (42). Likewise, the co-housing of zebrafish (*Danio rerio*) with differing microbial communities has been shown to lead to an emergent, group-specific gut microbiome due to the horizontal transmission of microbes (43). However, microbiome similarities and differences can also reflect the acquisition of specific microbes in response to shared environments or shared diets (39, 44).

Compared to many other wild animals, there exists a considerable amount of long-term, detailed field data on the plant and animal species composition of the diet of primates (45, 46), and, more recently, their gut microbiomes (47). While some wild primate species, such as geladas (*Theropithecus gelada*), chimpanzees (*Pan troglodytes*), and Verreaux’s sifakas, experience major changes in microbial composition over the course of a year (17, 21, 28), baboons (*Papio cynocephalus*) do not (48). These differences are likely linked to the degree of seasonal differences in diet. For example, in white-faced capuchins (*Carinotetraodon imitator*), the gut microbiome varies over the course of a year in seasonal dry forests (26), but it is relatively stable in aseasonal wet forests (49). Similarly, in certain human groups, temporal shifts in the gut microbiome are subtle, likely because their diets are less seasonally variable than wild nonhuman primates (50–54). Although seasonal food availability does modulate changes in gut microbiota composition in human populations (50, 52, 53), frequent social interactions among individuals also increase the similarity of the composition of their gut microbiome (55–57).

In this study, we assessed temporal and social dynamics in primate-microbe interactions by examining the gut microbiome of wild common marmosets (*Callithrix jacchus*) living in a Caatinga dry thorn scrub environment in northeastern Brazil. Common marmosets form small multimale multifemale social groups (mean group size is six individuals) with low levels of feeding competition (58, 59). Groups inhabiting the Caatinga are characterized by a single breeding female (60), who has the potential to produce two sets of twins per year. Offspring are cared for by all adult group members, especially males (60). The Caatinga biome in northeastern Brazil represents an extremely hot and dry environment. Mean monthly temperatures range from 25°C to 30°C, and annual rainfall for most of the region varies from 600 to 1,000 mm, with some areas averaging as low as 250 mm per year. Most rainfall occurs in a 3-month wet period; however, there is substantial year-to-year variation in rainfall, with the dry period lasting from 5 to 11 months (15, 61).

Common marmosets, and other primates inhabiting the Caatinga (*Alouatta caraya*, *Alouatta ululata*, *Callithrix penicillata*, *Sapajus libidinosus*, *Sapajus xanthosternos*, *Callicebus barbarabrownae*, and *Callicebus melanochir*), experience heat and water stress in addition to limited fruit availability (60, 62, 63). Like other mammals in Caatinga environments (62), common marmosets exhibit behavioral and morphological changes (increased time spent resting during periods of heat stress, smaller group sizes, and an increased surface area-to-body mass ratio compared to common marmosets in cooler and wetter habitats) (60, 64, 65). Their diet is dominated by the consumption of plant exudates (gums and saps) and insects throughout the year, supplemented with leaves, cactus flesh, flowers, fruits, spiders, earthworms, small vertebrates, and bird eggs (64, 66). This broad-based diet allows them to flexibly adapt to periods of low fruit availability. Invertebrates are a major source of lipids and proteins for common marmosets throughout the year, especially during the wetter periods (64). The contribution of individual insect orders to the diet, however, varies little across the year (64). Therefore, characterizing how both group and temporal variation impact the composition and function of the gut microbiome in this environment serves to provide a better understanding of animal phenotypic plasticity, health, and nutrition.

Our overall goal in this study was to assess the degree to which the composition and potential function of the gut microbiome of wild common marmosets change temporally in an extremely hot and dry environment characterized by minimal seasonal variation in rainfall. We aimed to determine whether changes in the gut microbiome represent an additional mechanism of plasticity to compensate for limited temporal variation in diet (64). We addressed the following questions: (i) is there temporal variation in plant, invertebrate, and vertebrate consumption in Caatinga common marmosets as indicated by a DNA metabarcoding analysis of consumed items? (ii) Is there evidence of temporal variation in gut microbiome composition and potential function? (iii) Do dietary differences between different periods of the year better account for variation in the gut microbiome than group membership? (iv) Do other factors, such as age and/or sex, shape gut microbiome composition and potential function in wild common marmosets? We hypothesized that microbiome composition and potential function would both differ among groups and vary temporally in association with diet composition.

## RESULTS

### Diet metabarcoding

**Fig 1:**
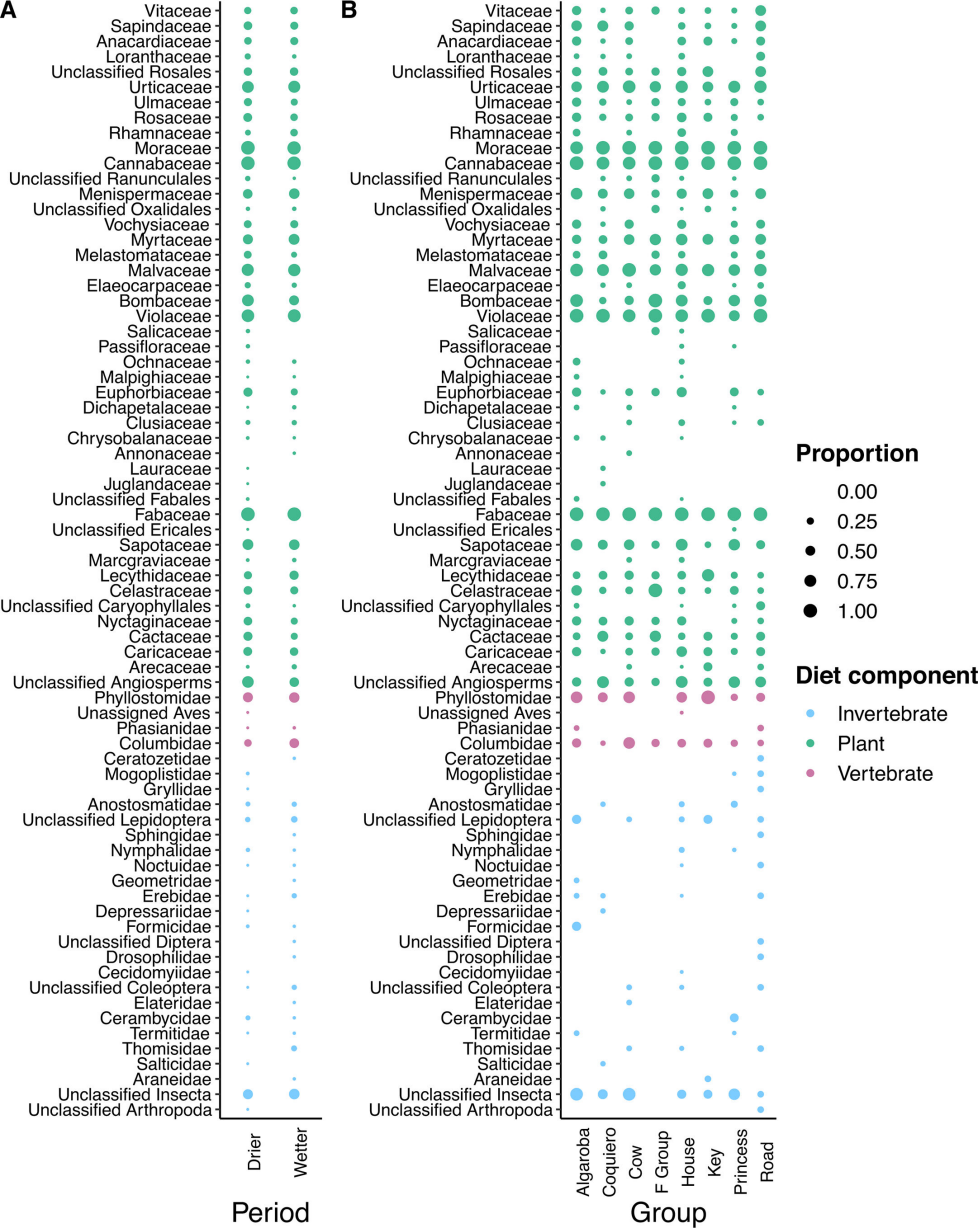
Proportion of samples (denoted by the size of the circle) in each time period or group containing DNA sequences assigned to arthropod (blue), plant (green), and vertebrate (purple) taxonomic groups. (**A**) Differences in diet between the drier and wetter periods. (**B**) Differences in diet between social groups.

We characterized the diet of common marmoset trapped at Baracuhy Biological Field Station (7°31′42″S, 36°17′50″W) in the state of Paraíba in northeastern Brazil using DNA metabarcoding. We amplified and sequenced the *trnL* (plants), *COI* (arthropods), and 16S rRNA (vertebrates) genes from fecal samples collected in 2015 and 2016 across both the wetter (February and March) and drier periods (July and August) (Table S1) from common marmosets belonging to eight neighboring social groups. We identified sequences from 8 orders and 19 families of arthropods, 3 orders and 3 families of vertebrates, and 17 orders and 38 families of plants in the feces of common marmosets. Many taxa were consumed in both the wetter and drier periods. However, 58% (11/19) of arthropod families and 13% (5/38) of plant families were only consumed during one part of the year (Fig. 1; Table S2). We found different levels of intergroup variation in the contribution of arthropods, vertebrates, and plant taxa to the diet of common marmosets. Whereas 92% (35/38) of identified plant families contributed to the diet of two or more groups, and 67% (2/3) of vertebrate families were shared between all eight groups, 74% (14/19) of arthropod families were identified in the feces of only one of the eight groups (Fig. 1; Table S2). Formicidae was only found in the Algaroba group; Salticidae and Depressariidae in Coqueiro; Elateridae in Cow; Cecidomyiidae and Geometridae in House; Araneidae in Key; Cerambycidae in Princess; and Drosophilidae, Sphingidae, Gryllidae, and Ceratozetidae in the Road group.

**Fig 2:**
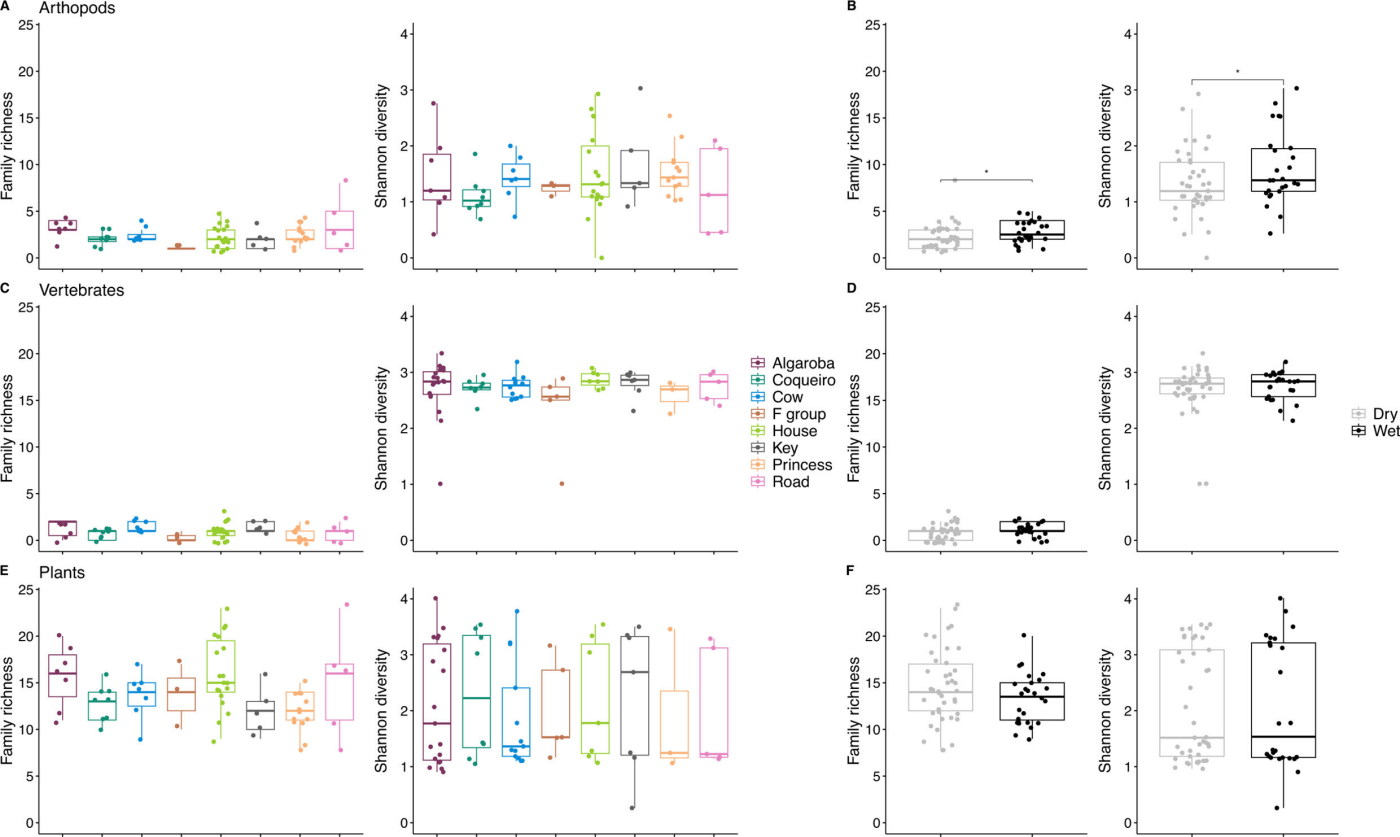
Richness and diversity of the invertebrate (**A and B**), vertebrate (**C and D**), and plant (**E and F**) sequences found in the feces of common marmosets across groups and time of year.

Species richness (i.e., number of species) of consumed arthropods was 23% higher and arthropod diversity 20% higher in the wetter period compared to the drier period (richness: *F* = 4.358, *P* = 0.041; Shannon: *F* = 6.030, *P* = 0.017) (Fig. 2; Table S3). The richness and diversity of vertebrates (including bats, birds, and/or bird eggs) and plants consumed did not vary by time period (Fig. 2; Table S3). In contrast, the richness of plant species in the diet varied significantly across groups (*F* = 2.530, *P* = 0.024), whereas the diversity of plant species did not (*P* > 0.05) (Table S3). The richness and diversity of the vertebrate and invertebrate components of the diet also did not vary between groups (Table S3). Importantly, we found that ethanol preserved arthropod DNA better than RNAlater as shown by a 16% increase in the Shannon diversity of arthropod taxa in fecal samples stored in ethanol (*F* = 9.042, *P* = 0.004) (Table S3). The effectiveness of these preservation buffers did not differ for vertebrate and plant DNA (Table S3).

We also examined relationships between diet composition and group, time period, sex, age classes, and preservative (Bray-Curtis and Jaccard indices) for each dietary component using permutational multivariate analysis of variances (PERMANOVAs). Group membership was not significantly associated with differences in the composition of any dietary component (all *P* > 0.05), even though group membership explained 11%−13% of the variation in diet composition (Table S4). Similar to the richness and diversity results above, there was a significant difference in the invertebrate composition of the diet between the wetter and drier periods (Bray-Curtis: *F* = 1.474, *R*^2^ = 0.026, *P* = 0.026; Jaccard: *F* = 1.577, *R*^2^ = 0.028, *P* = 0.007), but we did not find a difference in plant or vertebrate composition (Table S4).

### Gut microbiome composition

**Fig 3:**
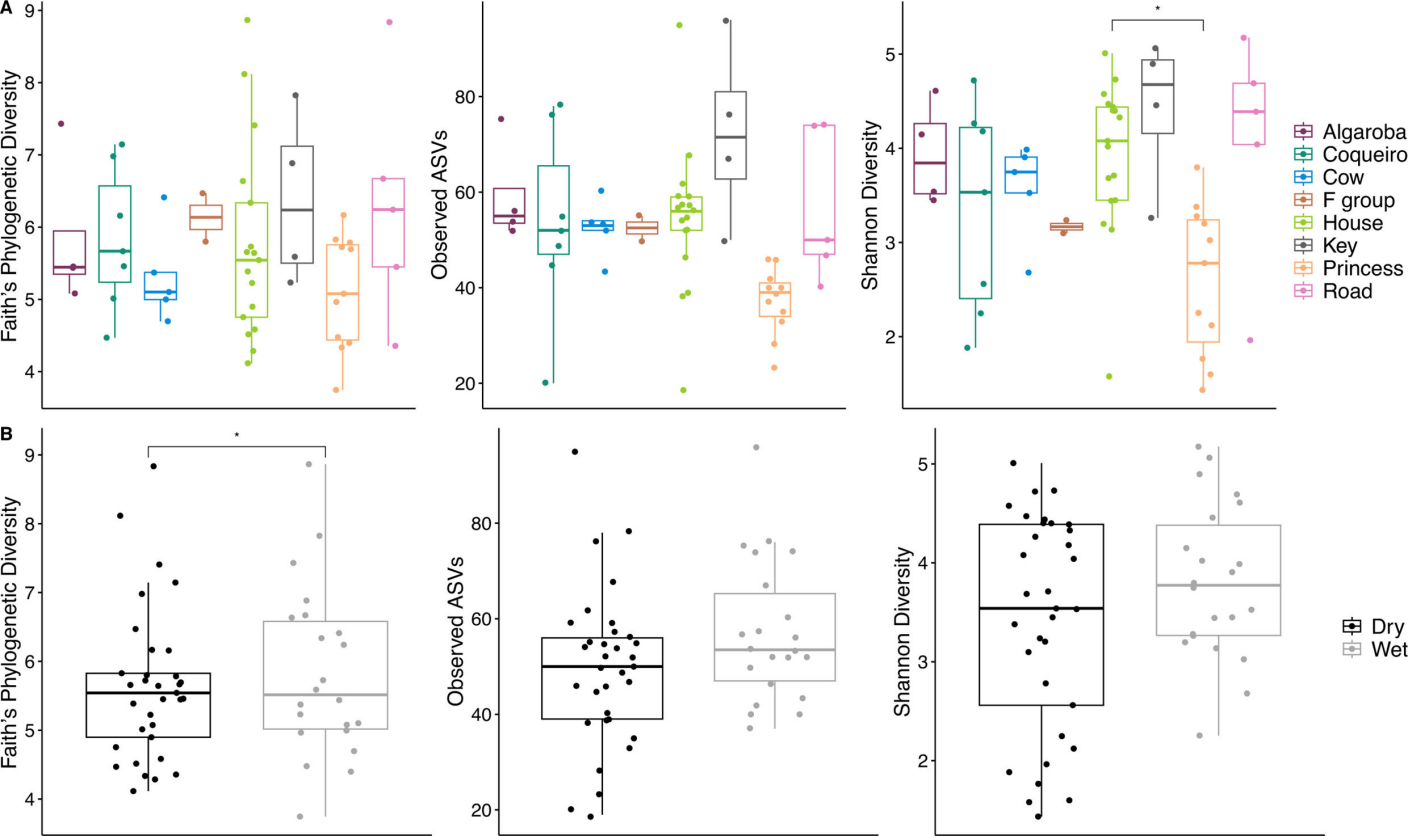
Comparisons of alpha diversity metrics—Faith’s PD, observed ASVs, and Shannon diversity—among groups (**A**) and time periods (**B**).

We assessed gut microbiome composition from the same fecal samples as above using 16S rRNA gene amplicon sequencing. Within-sample diversity—measured as Faith’s phylogenetic diversity (PD), observed amplicon sequence variants (ASVs), or Shannon diversity—did not differ consistently across metrics among groups, between time periods, or between preservatives (Fig. 3). Group predicted Shannon diversity (*F* = 2.599, *P* = 0.026) and Faith’s PD was 8.6% higher in the wetter period compared to the drier period (*F* = 4.229, *P* = 0.046). In addition, ethanol-preserved samples had a 5.0% higher Faith’s PD on average than RNAlater-preserved samples (*F* = 4.869, *P* = 0.033). Sex, age, and year of collection did not predict any diversity metric (all *P* > 0.05; Table S5).

**Fig 4:**
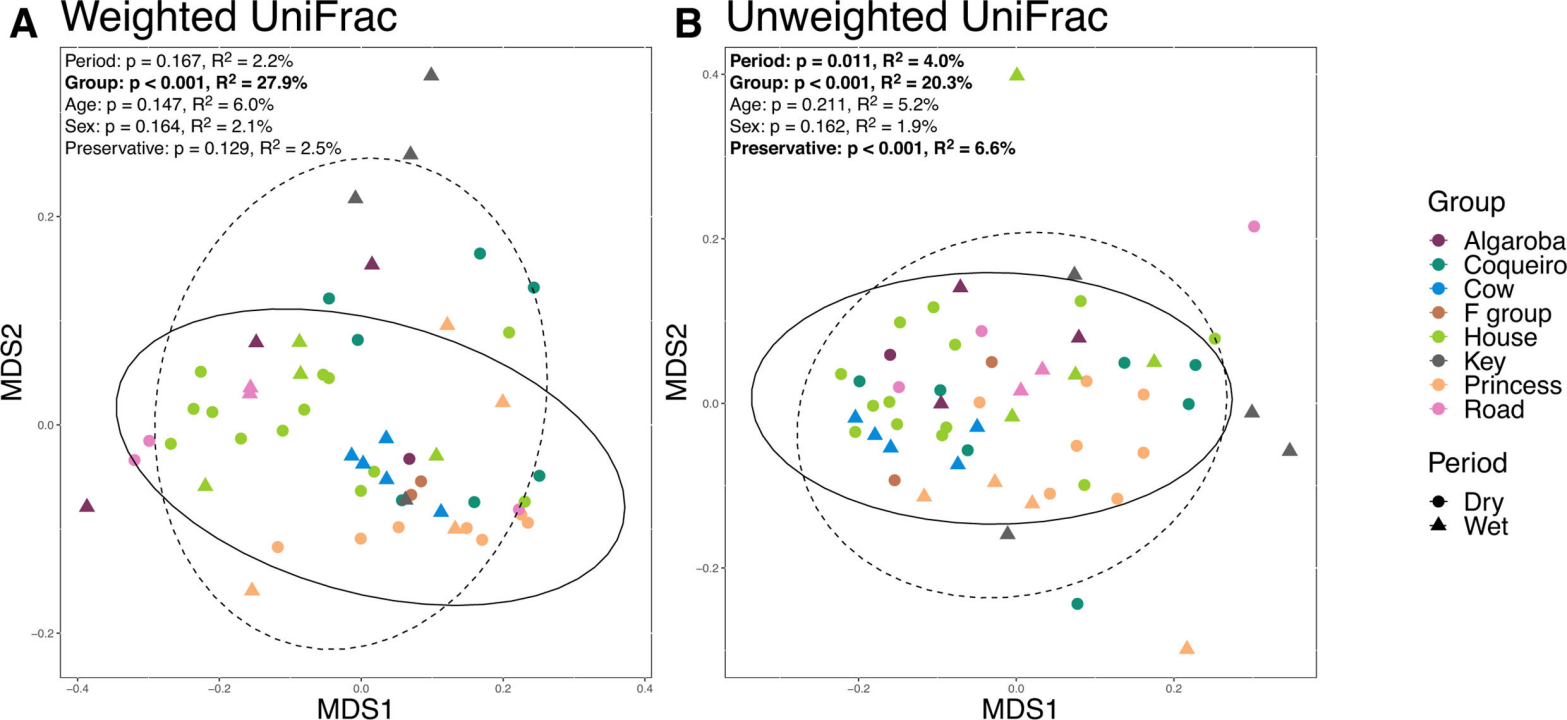
Ordination plot showing weighted (**A**) and unweighted (**B**) UniFrac distances. Colors denote group membership, and shape denotes drier vs wetter periods. 95% confidence ellipses encircle drier (solid line) and wetter (dashed line) period samples.

Group membership was the strongest predictor of both weighted and unweighted UniFrac distances of microbiome composition, although in both cases, it explained <28% of the observed variance (weighted: *F* = 2.954, *R*^2^ = 0.279, *P* < 0.001; unweighted: *F* = 2.005, *R*^2^ = 0.203, *P* < 0.001) (Fig. 4). Time period was significantly associated with microbiome composition, but only when examining unweighted UniFrac distances, and the strength of the effect was markedly lower than that of the group (*F* = 2.795, *R*^2^ = 0.040, *P* = 0.011) (Fig. 4). Age, sex, and year of collection were not predictive of gut microbiome composition (all *P* > 0.05; Table S6). Gut microbiome composition did differ between ethanol-preserved and RNAlater-preserved samples when examining the presence/absence of taxa (weighted UniFrac: *F* = 1.878, *R*^2^ = 0.025, *P* = 0.129; unweighted UniFrac: *F* = 4.560, *R*^2^ = 0.066, *P* = <0.001) (Fig. S1), but, again, the effect size was smaller than that of the group.

**Fig 5:**
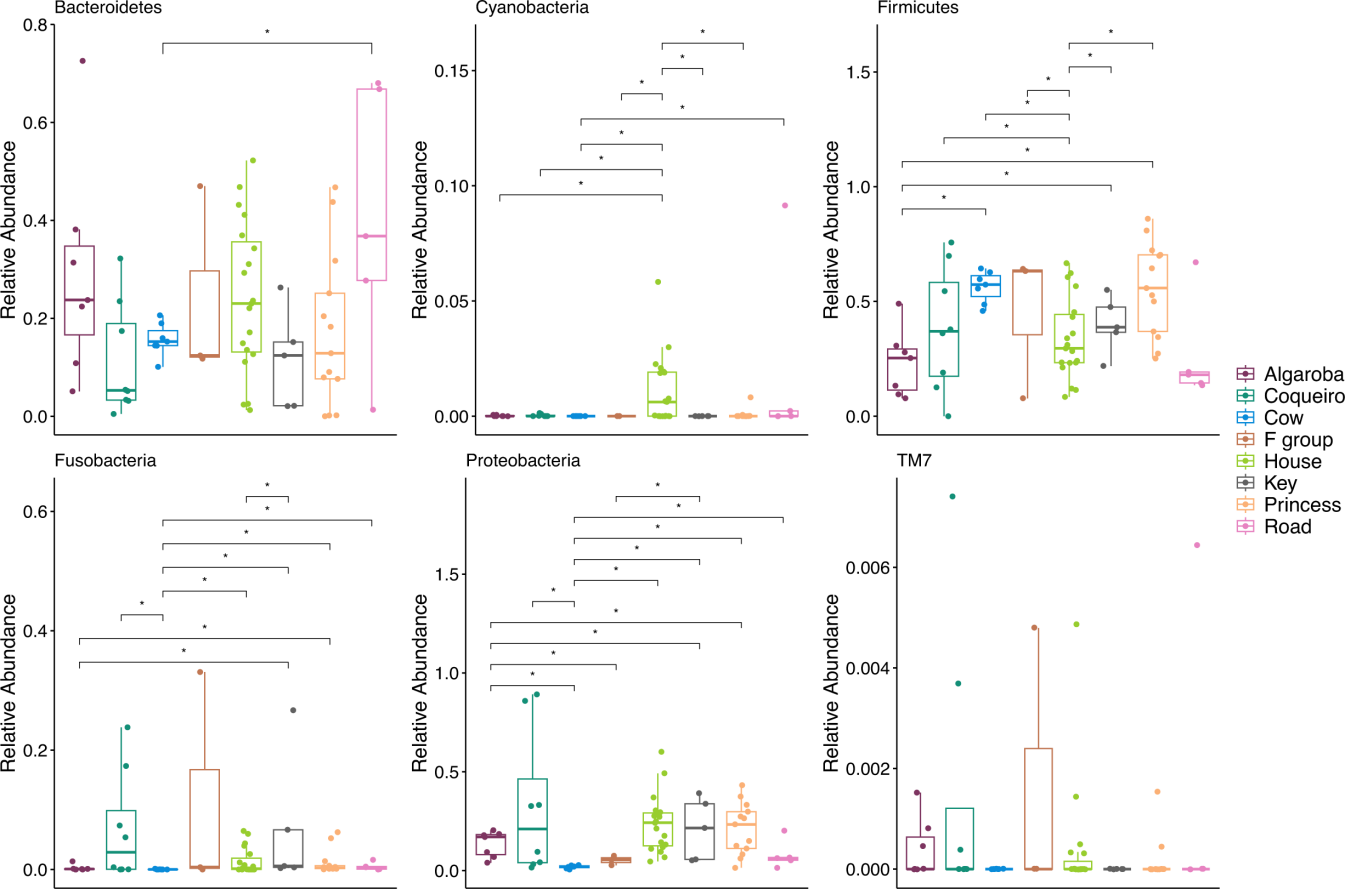
Phyla that differed in abundance among groups based on the global test of ANCOM-BC. Statistical significance bars denote pairwise among-group differences.

The abundance of many phyla, including Bacteroidetes, Cyanobacteria, Firmicutes, Fusobacteria, Proteobacteria, and TM7, differed among groups (all *q* < 0.05; Fig. 5; Table S7). Fusobacteria was more abundant in the wetter period than in the drier period (*W* = 3.130, *q* = 0.008). All other phyla did not differ in their abundance between time of year (all *q* > 0.05; Table S7). Similarly, multiple families, genera, and ASVs differed in abundance among groups, while only a few differed in abundance between the drier and wetter periods (Table S7).

### Gut microbiome function

**Fig 6:**
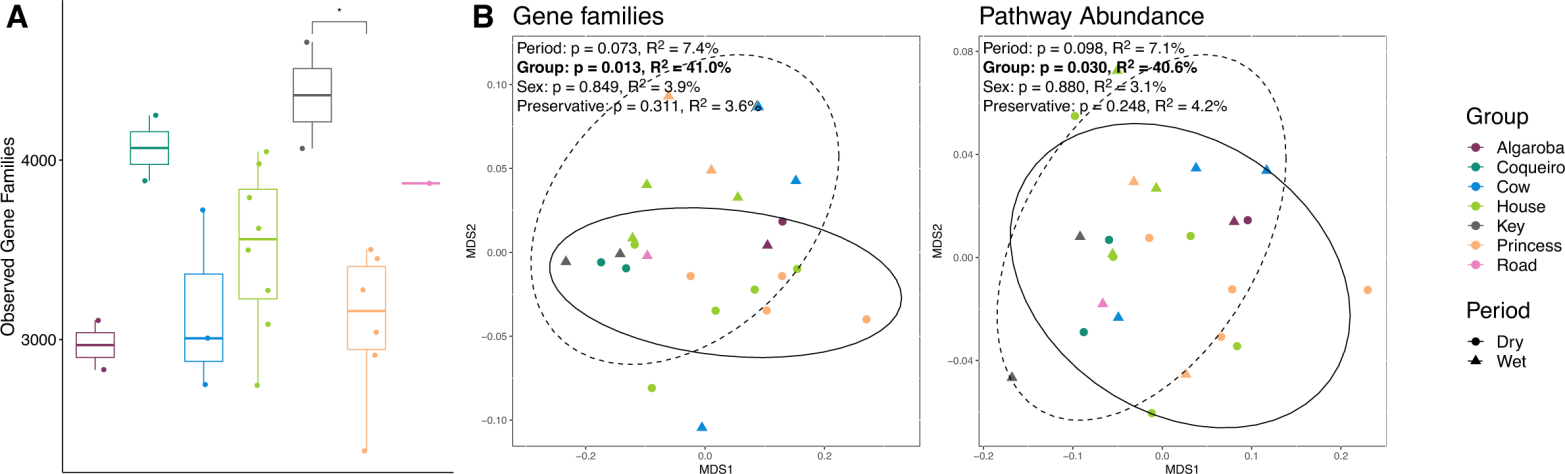
Alpha (**A**) and beta (**B**) diversity differences among groups for functional profiles. In the ordination plots, colors denote group membership and shape denotes drier vs wetter period. 95% confidence ellipses encircle dry (solid line) and wet (dashed line) samples.

A subset of samples (*n* = 24) was further analyzed for gut microbiome potential function using metagenomic sequencing. The number of observed gene families differed among groups (*F* = 3.226, *P* = 0.036, Fig. 6). Shannon diversity and evenness of gene families did not differ among groups (*P* > 0.05; Table S8). The number of observed gene families, Shannon diversity, and evenness of gene families did not differ between time periods, sexes, or collection years (*P* > 0.05; Table S8). For functional pathways, the number of observed pathways and the Shannon diversity and evenness of functional pathways did not differ among groups, time periods, or sexes (*P* > 0.05; Table S8). The collection year predicted the Shannon diversity of pathways (*F* = 4.942, *P* = 0.046), with 2016 having a lower pathway Shannon diversity than 2015, but the year did not predict the number of observed pathways or the evenness of pathways (*P* > 0.05; Table S8). Group was a strong predictor of the presence of gene families and functional pathways using Jaccard distances (gene families: *F* = 2.044, *R*^2^ = 0.410, *P* = 0.012; pathways: *F* = 2.049, *R*^2^ = 0.406, *P* = 0.034; Fig. 6). However, group did not predict gene family or functional pathway composition weighted for abundance using Bray-Curtis distances (gene families: *F* = 2.217, *R*^2^ = 0.074, *P* = 0.067; pathways: *F* = 2.152, *R*^2^ = 0.071, *P* = 0.097; Table S9). Time of year, sex, preservative, and year of collection did not predict gut microbiome potential function (all *P* > 0.05; Table S9).

The abundance of functional pathways did not differ by time of year, group, sex, or preservative (Table S10). A total of 1,989 KEGG orthogroups (KOs) differed in abundance among groups and 644 KOs also differed between sexes, whereas only 73 KOs differed in abundance between the wetter and drier periods. Eight KOs differed between ethanol- and RNAlater-preserved samples (Table S10). Butyryl-CoA CoA-transferase gene (BCoA) counts from RT-qPCR analyses did not differ between time periods, among groups, between sexes, or between sample preservation methods (all *P* > 0.05, Table S11).

### Diet-gut microbiome interactions

The invertebrate and the plant components of the diet were not correlated with microbial community composition (invertebrates: *r* = −0.135, *P* = 0.974; plants: *r* = −0.070, *P* = 0.844). Furthermore, diet composition was not a good predictor of microbial pathway abundance (invertebrates: *r* = −0.131, *P* = 0.820; plants: *r* = −0.171, *P* = 0.938) or microbial gene family abundance (invertebrates: *r* = −0.094, *P* = 0.724; plants: *r* = −0.172, *P* = 0.894). Similarly, we found only weak correlations between the richness and Shannon diversity of the diet and the composition and potential function of microbial communities (all *P* > 0.05) (Tables S12 and S13).

## DISCUSSION

In this study of wild common marmosets, we found that gut microbiome composition and potential function were most strongly associated with group membership and that time of year, diet, sex, preservative, and year of sample collection seemed to play minimal roles in shaping microbiome composition and diversity of the study population. Only social group was associated with differences in microbial gene family or pathway abundance. In contrast, we did not detect any associations between diet and gut microbiome composition or potential function. Additionally, the taxonomic composition of plants and invertebrates in the diet varied more across time of year than among groups. However, the DNA metabarcoding methods we used do not provide information on the amount of each plant and invertebrate taxon consumed; therefore, this result should be treated with caution. Nevertheless, diet appears to be a less important factor than group membership in structuring individual differences in the gut microbiome in this common marmoset population.

Species differences in rates of social interaction, group size and cohesion, group-based and temporal differences in diet, and habitat heterogeneity across the environment can affect the degree to which group membership or dietary patterns more strongly influence gut microbiome composition. Cohesive groups with frequent social interaction are expected to have high rates of horizontal or within-group microbial transmission (38, 40, 41). Alternatively, fine-scale ecological differences in the flora and fauna across each group’s home range can also result in high among-group variability in access to and the acquisition of environmental microbes (39, 44). While we do not have the necessary behavioral and ecological data to rigorously test a social behavior hypothesis vs a microhabitat difference hypothesis, it appears that both social and ecological factors play a role.

Caatinga common marmosets form small (four to nine group members) and highly cohesive social groups with low levels of feeding competition and high levels of social cooperation (58, 60). Based on DNA analyses, the taxa consumed and diet richness and diversity were found to vary among members of our eight study groups. For example, 14 of 19 arthropod families were uniquely found in the feces of a single group. In addition, the richness of plants consumed varied among groups. For instance, plant richness was significantly lower in the Princess group compared with the House group. However, plant species richness among the other six groups was not significantly different, and plant diversity did not differ among groups. Additionally, while group accounted for 11%–13% of the variation in invertebrate, plant, and vertebrate diet composition, the association between group and diet was not significant for any component of the diet. This stronger relationship between the gut microbiome and social group compared with between the gut microbiome and diet or seasonality is similar to the results in another primate species, *Propithecus verreauxi* (28). Multiple tests of diet-microbiome associations suggested there was no relationship between arthropod, vertebrate, and plant families consumed and the composition or potential function of the gut microbiome. Thus, it does not appear that diet alone, as reflected in DNA sequences present in feces, is driving group differences in microbiome composition.

We did find that the major temporal and group differences in diet were related to the specific taxa of arthropods consumed and an increase in exploiting cacti (plant family Cactaceae) during the drier period (41.5% drier period samples vs 28.6% wetter period samples). Cactaceae*,* which are common across the Caatinga landscape, provide food and water for common marmosets throughout the year. We found Cactaceae DNA in the fecal remains of all groups and across the entire year. Common marmosets consume cactus fruits, flowers, and flesh. This may reflect the high water content (77%–92%) present in cactus resources (67). Additionally, both increases and decreases in temperature appear to be more important drivers of patterns of activity and rest than food availability in Caatinga marmosets (68). Thus, factors, such as thermoregulation and water scarcity in addition to food availability, serve to shape the diet and, potentially, the gut microbiome of wild common marmosets.

There are limited data on temporal variation in the species and nutrient composition of insects consumed by wild primates (69–72). While the arthropod taxa being consumed by common marmosets appear to vary considerably across groups [17 of the 21 insect families identified were present in only one (*n* = 13) or two (*n* = 4) groups] and time of year, we are unable to assess variation in both the amount of individual insect taxa consumed and the nutritional content of the insect component of the common marmoset diet. In general, insects represent a high-quality source of protein and lipids for these small-bodied primates (69). Moreover, Fusobacteria, which is associated with prey consumption (29, 73, 74), differed in relative abundance across time periods, with the median relative abundance in the wetter period being >100% greater than in the drier period (Table S6). Carnivorous animals generally have relatively high abundances of Fusobacteria in their gut, compared with other animals. More specifically, Chinese alligators, whose rate of meat consumption varies across the year, have higher relative abundances of Fusobacteria when consuming more meat (29). In common marmosets, high relative abundances of Fusobacteria are associated with the wetter period of the year when insect consumption appears to be higher (64). Conversely, these same authors found that the specific insect orders consumed varied minimally across time periods.

In addition, 100% of samples in all groups during the entire year contained DNA from the plant family Fabaceae. Although we were not able to genetically identify the species of Fabaceae in the feces, based on behavioral observations of these groups, exudates of *Prosopis juliflora*, commonly known as algaroba, are consumed by these Caatinga common marmosets throughout the year and dominate their diet (64). Common marmosets exhibit adaptations of their mandibular incisors and canines that enable them to gouge holes in tree trunks and branches to elicit the flow of exudates (75). They also have an elongated and complexly folded cecum to facilitate the breakdown of difficult-to-digest carbohydrates that may be present in exudates (76). Moreover, common marmosets are reported to frequently scent mark (using their suprapubic gland) and urine mark recently gouged exudate holes (77). Other group members visit and feed at these marked sites, and this may represent a mechanism of within-group bacterial exchange. Overall, it appears that several elements of the marmoset diet are common across all groups and, to some extent, time of year (i.e., *P. juliflora* exudates, cacti, and insects), while the ingestion of other food items varied based on their availability in the group’s home range.

While the lack of correlation between diet and microbiome composition or potential function and the absence of a temporal pattern in the microbiome was contrary to our predictions, it may offer important insight into how the microbiome responds to constant vs changing environmental conditions. Viewing the Caatinga as an aseasonal environment in terms of rainfall (very limited rainfall across the entire year) may serve to explain the patterns we have found. Based on climatological data collected in the nearby town of Cabaceiras (5 km from the field site), the average monthly rainfall across the 2-year study period was 14.4 mm (range: 0–52 mm) (13). During the wetter period, monthly rainfall averaged 22 mm in 2015 and 19 mm in 2016, whereas monthly rainfall during the drier period averaged 9 mm in 2015 and 7 mm in 2016 (13).

Variability in the gut microbiome likely contributes to adaptive phenotypic plasticity in the host (78, 79), but phenotypic plasticity occurs within defined limits in response to external or internal stimuli (80–82). If a change to the gut microbial community does not increase digestive efficiency or if the variability in the environment is not predictable, there may be limited selective pressure for microbiome compositional variation. Additionally, there are conditions under which the gut microbiome does not buffer or benefit the host, such as extreme temperatures, anthropogenically disturbed habitats, and conditions of low nutrient availability (2, 83–85). For instance, high ambient temperatures were found to disrupt the gut microbiome of the red-backed salamander (*Plethodon cinereus*), reducing its digestive efficiency and microbiome diversity (85). Likewise, red colobus (*Procolobus gordonorum*) and black howler monkeys (*Alouatta pigra*) living in highly anthropogenically disturbed environments have gut microbiomes that are less diverse than their conspecifics inhabiting less disturbed habitats (83, 84). The Caatinga environment at our field site is best characterized as an aseasonal, hot, dry, and highly disturbed environment, and the gut microbiome of Caatinga common marmosets may, therefore, have limited plasticity or temporal variability.

Alternatively, the specific nutrients (amount of dietary fiber, protein, lipids, carbohydrates, and micronutrients) contained in the foods consumed by Caatinga common marmosets may vary minimally across the year. For primates that exploit an exudate- and/or insect-heavy diet, day-to-day microbiome plasticity may be more important than long-term variability to compensate for variation in nutrient consumption (48). This is due to the fact that the insects consumed by common marmosets may not represent a homogenous resource. The most commonly consumed invertebrates in our study include members of Lepidoptera (moths and butterflies: 28.4% of samples), Orthoptera (grasshoppers and crickets: 13.4% of samples), Coleoptera (beetles: 13.4% of samples), and Araneae (spiders: 9.0% of samples). The macronutrient content of the invertebrates consumed by primates can vary markedly (69, 71, 86, 87). For example, insects consumed by red-tailed monkeys (*Cercopithecus ascanius*) varied in protein content from ca. 57% to 78% protein (as a percentage of dry matter) and ca. 7% to 17% fat (69). Therefore, daily and interindividual variation in nutrient consumption and microbial exchange among group members may outweigh temporal variation in exudate-insect feeding primates, such as common marmosets.

Finally, our data suggest that the gut microbiome of wild common marmosets is generally similar in overall composition to that of a taxonomically close relative, the wild saddleback tamarin (*Leontocebus weddelli*), which inhabits a tropical rainforest environment (88). Saddleback tamarins consume fruits, exudates, and invertebrates, and their microbiome is dominated by Bacteroidetes (62%), Firmicutes (21%), and Proteobacteria (11%). Although saddleback tamarins also live in small, cohesive social groups (group size averages five to eight individuals), there was no evidence that group membership was associated with gut microbial community structure or potential function (88), as was the case for common marmosets. In this regard, it is possible that given both their extremely small home range (1–5 ha) and the heterogeneity of their Caatinga environment (89), different groups of common marmosets encounter different patterns of resource distributions than saddleback tamarins encounter across their larger, less anthropogenically disturbed home range (20–40 ha) and tropical rainforest habitat (88).

### Conclusions

In conclusion, we found strong differences in gut microbiome composition and potential function among wild common marmosets living in neighboring social groups, but not across different periods of the year. Future studies that systematically test how individual host species factors contribute to the strength of environmental or social influences on gut microbiome variability will help clarify the principles underlying host-associated microbial community dynamics. In particular, comparative studies across multiple groups of closely related species with divergent behavior or dietary patterns would allow us to assess the range of variation in gut microbiome composition and potential function. Moreover, studies of multiple populations of a single species across their range would provide valuable data on the potential range of variation of the gut microbiome within a host species across environments. In addition, obtaining data on the diet and nutritional content of individual foods consumed by Caatinga common marmosets should be particularly informative in explaining our findings. In light of the growing recognition of limitations in the ability of the gut microbiome to contribute to host phenotypic plasticity, we hope that future studies will be specifically designed to test when the gut microbiome is contributing to host phenotypic plasticity and what the limits and costs of microbial contributions may be.

## MATERIALS AND METHODS

### Study site and sample collection

We conducted common marmoset trapping and DNA sample collection at Baracuhy Biological Field Station (7°31′42″S, 36°17′50″W) in the state of Paraíba in northeastern Brazil, a 400 ha privately owned working farm and field station. The site is classified as a hot semiarid climate and is characterized by anthropogenically disturbed thorn scrub vegetation. Daytime temperatures may exceed 35°C for at least 7 months of the year and rainfall averages 337 mm per year, making this one of the driest habitats occupied by nonhuman primates (14, 60, 63, 65). Climate data collected over an 85-year period indicates no rainfall in some years (14). The mean temperature is variable across the year—in the wetter period, the mean maximum temperature is 29.1°C and the mean minimum temperature is 19.3°C, while the mean maximum temperature during the drier period is 31.3°C and the mean minimum temperature is 20.8°C (64).

We collected fecal samples in 2015 and 2016 during both the wetter (February and March) and drier periods (July and August) (four total sampling periods: 2015 wet period, 2015 dry period, 2016 wet period, and 2016 dry period) from common marmosets belonging to eight social groups. We trapped, marked, and released marmosets unharmed as part of a comprehensive study of decision-making, social foraging, and reproductive strategies (58, 60, 63, 90). Marmosets were anesthetized between 30 min and 3 hours of capture, and we retrieved fecal samples from collection pans set under individual compartments of the traps at the time of anesthetization. Samples were stored in sterile tubes filled with either RNAlater or 95% ethanol. Our sample collection protocol changed over the course of the project, so samples were preserved either in RNAlater or in 95% ethanol, but not both. We determined age (based on dental eruption and dental wear) and sex for all individuals, and female reproductive status (pregnancy/non-pregnant; lactating/non-lactating) (60, 63). We fitted all adult and subadult individuals with a uniquely colored beaded collar for identification in the field. We could not positively identify all marmosets when they were trapped (some collars may have fallen off between collection periods). Each marmoset was only sampled once in a given collection period, but we cannot know whether or not the same marmoset was sampled in more than one collection period. Samples were stored and shipped at ambient temperature. Upon arrival in the United States, samples were stored at −80°C prior to DNA extraction.

### DNA extraction

We extracted DNA from 67 fecal samples (drier period: *n* = 41, wetter period: *n* = 26) following established protocols (91). Briefly, samples were extracted with a Qiagen PowerSoil Kit with the following modifications: (1) we incubated samples at 65°C for 15 min after the addition of solution C1 and prior to bead beating; and (2) we incubated samples at room temperature for 5 min with solution C6 pre-warmed to 65°C prior to centrifugation. We performed extraction negatives to control for contamination during extraction using molecular-grade water instead of a sample.

### Diet metabarcoding and analysis

Many primate species regularly consume invertebrates, but observations of invertebrate consumption often lack detail, as invertebrate taxa are difficult to identify as they are being consumed, especially by small monkeys (69, 92–95). Thus, we used DNA metabarcoding as an alternative method to identify the invertebrates consumed by common marmosets during different periods of the year (49, 96, 97).

We amplified all samples using a Fluidigm Access Array system. We targeted plants using the trnLg-trnLh primer pair (98), arthropods using the ArtF11-ArtR17 primer pair (97), and vertebrates using the L2513-H2714 primer pair (99). We performed PCR using established protocols (96). We sequenced samples on the Illumina NovaSeq platform using a 2 × 250 bp SP flowcell at the Roy J. Carver Biotechnology Center at the University of Illinois Urbana-Champaign.

Sequencing yielded 118,440,852 reads (mean ± sd: 1,741,777 ± 822,636 per sample). We used Obitools3 (version 3.0.0b33) to pair, quality-filter, and assign taxonomy to reads (100). We constructed reference databases for arthropods, plants, and vertebrates from the EMBL nucleotide sequence database (downloaded 17 August 2020) using ecoPCR as implemented in Obitools3 (101).

### 16S rRNA gene amplification, sequencing, and analysis

We amplified the V4–V5 region of the 16S rRNA gene for all samples with a two-step PCR protocol using the 515F-926R primer set (102, 103) following established protocols (104). We performed PCR negatives to control for contamination during PCR using molecular-grade water in place of a DNA template. We normalized samples using a SequalPrep Normalization Plate. We sequenced samples, extraction negatives, and PCR negatives using 2 × 300 bp reads on the Illumina MiSeq V4 platform at the University of Illinois Chicago Genome Research Core.

We obtained 2,350,299 sequences (mean ± sd: 35,0279 ± 4,382 per sample). We used the DADA2 workflow in QIIME2 (version 2.2021.2) to trim, quality-filter, and denoise raw 16S rRNA gene sequences into amplicon sequence variants. Denoising resulted in 1,129,762 ASVs (mean ± sd: 16,862 ± 5,995 ASVs per sample). We classified the representative sequences using a naive Bayesian classifier trained on the V4–V5 region of the 16S rRNA gene in the Greengenes 13_8 database. We filtered ASVs identified as mitochondrial or chloroplast sequences from the data set prior to downstream analyses. We calculated alpha and beta diversity indexes in QIIME2 after rarifying samples to 8,956 reads to allow for even sampling across the data set. We excluded two samples from the drier period (CJAC4 and CJAC8) due to low read counts.

### Metagenomic library preparation, sequencing, and analysis

Most studies have relied on 16S rRNA gene sequencing and functional predictions based on compositional data to examine temporal changes and group differences in the gut microbiome, with few exceptions (26, 32, 38, 105). To understand the host-relevant functional consequences of compositional changes, we used metagenomic sequencing to assess changes in the potential function of the gut microbiome as a whole.

We selected 24 from the 69 samples above for metagenomic library preparation. We chose 12 adult wetter period (6 female and 6 male) and 12 adult drier period samples (6 female and 6 male) that had adequate DNA concentration and quality to use for library construction. We used a NuGen Celero DNA-Seq Kit with enzymatic fragmentation to construct metagenomic libraries following the manufacturer’s protocol. We performed fragmentation at 25°C for 10 min to select for a target insert size of 400–500 bp. We barcoded samples with unique dual indexes. Libraries were size selected to 300–500 bp using PippinPrep and pooled based on the results of an Illumina MiniSeq run. We sequenced samples on the Illumina NovaSeq platform using a 2 × 150 bp SP flowcell at the University of Illinois Chicago Genome Research Core.

Sequencing yielded 474,017,905 reads (mean ± sd: 19,750,746 ± 16,379,608 per sample). We trimmed and quality-filtered raw sequences using KneadData (version 0.8.0). We cleaned forward and reverse reads separately for each sample and concatenated them prior to further analysis. We used HUMAnN2 (version 2.8.2) (106) for microbial functional profiling. We calculated MetaCyc reaction pathway abundances and gene family abundances collapsed into KEGG Orthogroups in HUMAnN2.

### BCoA gene RT-qPCR and analysis

We used RT-qPCR to detect changes in butyrate gene abundance to examine temporal variation in the contribution of the gut microbiome to host energy sources. We assessed the abundance of butyrate-producing bacteria by RT-qPCR of the butyryl-CoA CoA-transferase gene (BCoA) using the BCoATscrF-BCoATscrR primer pair (107). We assayed all samples in triplicate in 10 µL reaction volumes containing 2.5 µL gDNA, 5 µL of 2× Fast SYBRGreen Master Mix, 0.25 µL of each primer at 10 µM concentration, and 2 µL of nuclease-free water. We ran reactions on a ViiA7 Real-Time PCR System under the following conditions: 2 min at 50°C, 20 s at 95°C, and 40 cycles of 1 s at 95°C, 20 s at 58.5°C, and 30 s at 72°C. We verified the PCR product on an agarose gel and used the automatic threshold cycle (Ct) number generated after each run for all analyses.

### Statistical analysis of DNA metabarcoding and microbiome data

We performed all statistical analyses in R (r-project.org, version 4.2.1) (108). We assessed diet diversity by calculating species richness and Shannon diversity using the vegan package (version 2.6-4). We calculated Jaccard (community dissimilarity based on presence-absence) and Bray-Curtis (community dissimilarity taking into account taxa abundance) distances for diet compositional data using the vegan package (109). We used linear regression to examine the effect of time of year and group on diet diversity. In addition, we used linear regression to examine the effect of time period, group, age, and sex on gut microbiome alpha diversity and BCoA gene counts using the nlme (version 3.1-164), multcomp (version 1.4-25), and car packages (version 3.1-2) (110–112). We used PERMANOVA to assess associations between time period, group, age, and sex on diet composition, gut microbial community composition, and potential function using the vegan package. We detected microbial phyla, families, genera, and ASVs that were differentially abundant between time periods and among groups using an analysis of the composition of microbiomes with bias correction and adjustment for multiple comparisons using the Benjamini-Hochberg false discovery rate, as implemented in the ANCOM-BC package (version 1.6.4) (113).

We detected pathway abundances and gene family abundances that were differentially abundant between time periods and among groups using generalized linear models with a negative binomial distribution in the glmmTMB package (version 1.1.8) (114) and adjustment for multiple comparisons using Benjamini-Hochberg false discovery rate, as implemented using the fdrtool package (version 1.2.17) (115). We included preservative (ethanol vs RNAlater) in all models to control for the effect of differing storage conditions on DNA preservation (Tables S1 to S8). We also examined the effect of year to account for year-to-year variation in annual rainfall on overall microbiome composition and potential function (Tables S2, S3, S6, and S7). Additionally, some groups had access to human foods and/or were in close proximity to domestic animals; therefore, we also ran models using those variables to determine if they were a source of variation (Tables S3, S4, S6, and S7). Samples from individuals with unknown age and/or sex were excluded from full models.

### Microbiome diet associations

We examined diet-microbiome correlations by using Mantel tests in the vegan package in R to compare Bray-Curtis dissimilarity matrices for each dietary component and both microbiome composition (from 16S rRNA gene sequencing data) and functional potential (from metagenomic sequencing data). Additionally, we used Spearman correlations to test for associations between the richness and Shannon diversity of each dietary component and microbiome composition and potential function. We did not test correlations between microbiome and vertebrate diet composition due to a high number (21 of 67) of fecal samples containing no vertebrate DNA.

## Data Availability

Sequences are available in the Short Read Archive (https://www.ncbi.nlm.nih.gov/sra) under BioProject PRJNA1021612. All metadata used in the analyses and code for all analyses can be found on GitHub (https://github.com/emallott/Caatinga_marmosets).

## SUPPLEMENTAL MATERIAL

Figure S1 msphere.00233-24-s0001.docx Comparisons of alpha diversity metrics between ethanol-preserved and RNALater-preserved samples.

Supplemental Tables msphere.00233-24-s0002.xlsx Tables S1 to S13.

ASM does not own the copyrights to Supplemental Material that may be linked to, or accessed through, an article. The authors have granted ASM a non-exclusive, world-wide license to publish the Supplemental Material files. Please contact the corresponding author directly for reuse.
