# Supplementary material for: Group membership, not diet, structures the composition and functional potential of the gut microbiome in a wild primate
Source: mSphere. 2024 Jun 28;9(7):e00233-24. doi: 10.1128/msphere.00233-24 (PMC11288025; doi:10.1128/msphere.00233-24)
Supplement: Figure S1 — Comparisons of alpha diversity metrics between ethanol-preserved and RNALater-preserved samples. [file msphere.00233-24-s0001.docx]

**
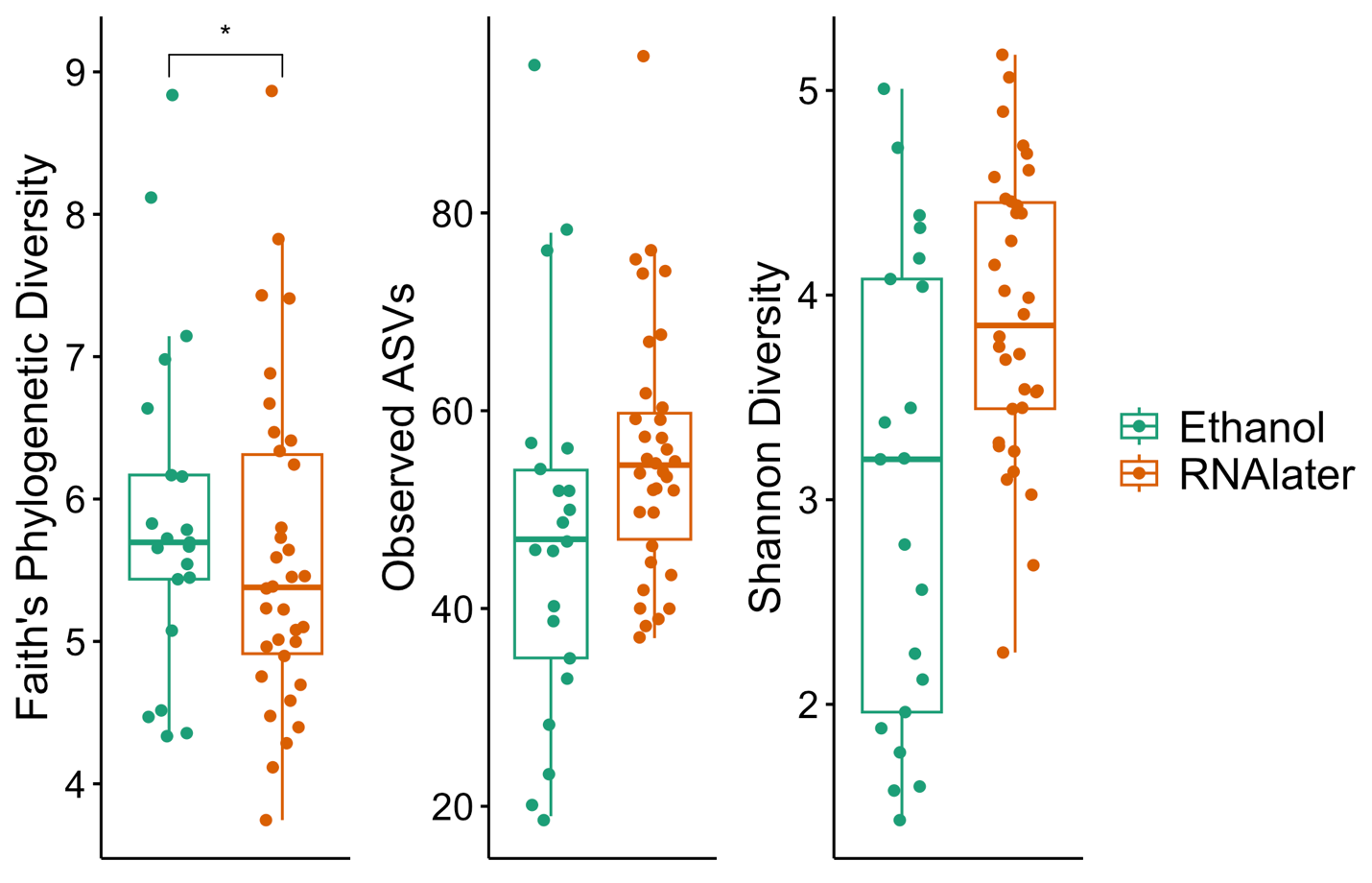
**

**Figure S1.** Comparisons of alpha diversity metrics – Faith’s PD, observed ASVs, and Shannon diversity – between ethanol-preserved and RNALater-preserved samples.
